# Review: The Important Bacterial Zoonoses in “*One Health*” Concept

**DOI:** 10.3389/fpubh.2014.00144

**Published:** 2014-10-14

**Authors:** Leon Cantas, Kaya Suer

**Affiliations:** ^1^Norwegian Private Veterinary Services, MicroLab, Hammerfest, Norway; ^2^Department of Medical Microbiology, Faculty of Medicine, Near East University, Nicosia, Cyprus; ^3^Department of Infectious Diseases and Clinical Microbiology, Faculty of Medicine, Near East University, Nicosia, Cyprus

**Keywords:** zoonoses, human, animal, bacterial diseases

## Abstract

An infectious disease that is transmitted from animals to humans, sometimes by a vector, is called zoonosis. The focus of this review article is on the most common emerging and re-emerging bacterial zoonotic diseases. The role of “*One Health*” approach, public health education, and some measures that can be taken to prevent zoonotic bacterial infections are discussed.

**Key points:**

- A zoonotic bacterial disease is a disease that can be very commonly transmitted between animals and humans. Global climate changes, overuse of antimicrobials in medicine, more intensified farm settings, and closer interactions with animals facilitate emergence or re-emergence of bacterial zoonotic infections.
- The global “*One Health*” approach, which requires interdisciplinary collaborations and communications in all aspects of health care for humans, animals, and the environment, will support public health in general.
- New strategies for continuous dissemination of multidisciplinary research findings related to zoonotic bacterial diseases are hence needed.

## Introduction

Zoonotic diseases are those infections that can be transmitted between animals and humans with or without vectors. There are approximately 1500 pathogens, which are known to infect humans and 61% of these cause zoonotic diseases (1). The unique dynamic interaction between the humans, animals, and pathogens, sharing the same environment should be considered within the “*One Health*” approach, which dates back to ancient times of Hippocrates (2, 3).

Bacterial zoonotic diseases can be transferred from animals to humans in many ways (4): (i) The transfer may occur through animal bites and scratches (5); (ii) zoonotic bacteria originating from food animals can reach people through direct fecal oral route, contaminated animal food products, improper food handling, and inadequate cooking (6–8); (iii) farmers and animal health workers (i.e., veterinarians) are at increased risk of exposure to certain zoonotic pathogens and they may catch zoonotic bacteria; they could also become carriers of the zoonotic bacteria that can be spread to other humans in the community (9); (iv) vectors, frequently arthropods, such as mosquitoes, ticks, fleas, and lice can actively or passively transmit bacterial zoonotic diseases to humans. (10); (v) soil and water recourses, which are contaminated with manure contains a great variety of zoonotic bacteria, creating a great risk for zoonotic bugs and immense pool of resistance genes that are available for transfer of bacteria that cause human diseases (11, 12).

Bacterial zoonotic infections are one of the zoonotic diseases, which can, in particular, re-emerge after they are considered to be eradicated or under control. The development of antimicrobial resistance due to over-/misuse of antibiotics is also a globally increasing public health problem. These diseases have a negative impact on travel, commerce, and economies worldwide. In most industrialized countries, antibiotic resistant zoonotic bacterial diseases are of particular importance for at-risk groups such as young, old, pregnant, and immune-compromised individuals (13).

Almost 100 years ago, prior to application of hygiene rules and discovery of neither vaccines nor antibiotics, some bacterial zoonotic diseases such as bovine tuberculosis, bubonic plague, and glanders caused millions of human deaths. The spread and importance of some bacterial zoonoses are currently globally increasing. That is precisely why most of the developing countries are sparing more resources for a better screening of animal products and bacterial reservoirs or vectors for an optimal preventative public health service (14).

Improvements in surveillance and diagnostics have caused increased recognition of emerging zoonotic diseases. Herein, changes in our lifestyles and closer contacts with animals have escalated or caused the re-emergence of some bacterial infections. Some studies lately have revealed that people have never been exposed to bacterial zoonotic infection risks as high as this before (15). It is probably due to closer contact with adopted small animals, which are accepted and treated as a family member in houses. On the other hand, more intensified animal farms, which have a crucial role in the food supply, are still one of the greatest sources of food-borne bacterial zoonotic pathogens in today’s growing world (4, 8).

People who have closer contact with large numbers of animals such as farmers, abattoir workers, zoo/pet-shop workers, and veterinarians are at a higher risk of contracting a zoonotic disease. Members of the wider community are also at risk from those zoonoses that can be transmitted by family pets.

The immune-suppressed people are especially at high risk for infection with zoonotic bacterial diseases. People can be either temporarily immuno-suppressed owing to pregnancy, infant age, or long-term immuno-suppressed as a result of cancer treatment or organ transplant, diabetes, alcoholism or an infectious disease (i.e., AIDS).

This manuscript reviews the most common bacterial zoonoses and practical control measures against them.

## Companion Animal-Borne Zoonoses

Companion animals are increasingly treated as family members, and pets have many bacteria that may infect their owners. The human population of the European Union (EU) was approximately 500 million^1^ in 2012. The number of pet owning households was estimated at around 70 million in 2010^2^.

The most commonly suffered zoonotic bacterial infections in humans are transmitted via animal bites and scratches. Various dog breeds have been characterized for their role in killing dog bite attacks, such as pit bull breeds, malamutes, chows, rottweiler, huskies, German shepherds, and wolf hybrids (16–18). In USA, pit bull breeds accounted for almost half of the dog bite-related zoonotic infections, three times more than German shepherds (17). The oral cavity of healthy dogs and cats contains hundreds of different pathogenic bacteria including *Pasteurella sp*. (19). Only 20% of dog bites get infected overall compared with 60% in cats. There are 10 times higher *Pasteurella multocida* infection risks after a cat bite than a dog bite (20, 21). *P. multocida* infected bite wounds appear usually within 8 h.

It is estimated that approximately 20% of animal bites or scratches get infected in humans (5). Bacterial culturing from pet bite infections in humans is found to be smilar to the oral microbiota of the pets. Infections in dog bite wounds are usually dominated by aerobic bugs: *P. multocida* (50%), alpha-hemolytic *Streptococcus* (46%)*, Staphylococcus* (46%)*, Neisseria* (32%), *and Corynebacterium* (12%). However, following anaerobic bacteria are also isolated from infected wounds: *Fusobacterium nucleatum* (16%), *Prevotella heparinolytica* (14%), *Propionibacterium acnes* (14%), *Prevotella intermedia* (8%), and *Peptostreptococcus anaerobius* (8%) (22).

Normal human skin bacteria or other environmental microorganisms are scarcely isolated from infected wounds in bitten person (22–24). Usually, infection occurs within 8–24 h after the animal attack, with variable pain on the site of the injury. The cellulitis might be followed by discharge that contains pus, which can sometimes be foul-smelling. Immuno-suppressed patients with diabetes or liver dysfunction are frequently predisposed to develop serious infections after animal bites. In those cases, they may develop bacteremia faster and pass away in a shorter period of time (5). A penetrating bite close to the joints and bones may cause septic arthritis and osteomyelitis. Knowing the microbial composition of dental plaque biofilm formation in pets’ mouth is a key factor in wound chronicity in humans (5, 25).

Cat-scratch disease is a clinical syndrome that has been reported in people for over 100 years. Yet, the etiological agent *Bartonella henselae*, which was transmitted by cat scratches and bites, was only identified in 1992 (26). However, contact with cat saliva on broken skin or sclera can also cause Bartonellosis. A person who has had a cat scratch may show papules and pustules at the site of injury (the first initial sign). The disease may progress with a chronic non-healing wound, fever (sometimes), weak regional lymph circulation, and abscession. Cat owners and veterinarians are most at risk (27). Systematic medical treatment is usually needed in people with suppressed immune systems. Otherwise, encephalopathy, osteomyelitis, and granulomatous conjunctivitis might develop.

Horses and humans have always shared a close relationship due to recreation, sporting, and occupational reasons, for over thousands of years. In Europe, the number of horses per capita remained relatively stable during the past decade. Germany and Great Britain have the largest horse populations in the EU, whereas Sweden has the highest number of horses per capita. The frequency of infected horse bite wounds is estimated to be 3–5% in Europe (28, 29). However, it has been roughly estimated that the horse bites account for as high as 20% of overall animal bites in Turkey, which comes after dog bites (70%) (30). More extensive muscle damage may develop in most of the horse attacks, which is different from small animal bites. A mixture of aerobic and anaerobic organisms has been isolated from horse bites in humans, which are frequently dominated by *Actinobacillus lignieresii* (31, 32). *Escherichia coli* and *Bacteroides* species have also been isolated from foul-smelling infections and pus drainage after horse bites in humans (33).

Infectious diarrhea in companion animals is caused by *Salmonella sp., Escherichia coli, Shigella sp.*, and *Campylobacter sp*. can also be transmitted to people through fecal oral route. It is difficult to estimate the distribution of these ubiquitous microorganisms. But it is known that they can be isolated from many healthy animals, which can be shed in their feces for long periods of time. Campylobacteriosis were the most frequently reported zoonotic bacterial diseases in 2009 among the EU member countries in humans (34). Like many other enteropathogens, they can cause gastroenteritis (diarrhea, vomiting), headaches, and depression, sometimes even leading to death. It is obvious that raw food diets for pets dramatically increase the risk of human exposure to such zoonotic bacterial enteropathogens, which cause gastrointestinal diseases.

Although pet birds, also called songbirds (e.g., canaries, finches, sparrows) and psittaciformes (e.g., parrots, parakeets, budgerigars, love birds) are a small fraction of adopted pets, they are widely popular in Europe and they are potential carriers of zoonotic diseases (35). Some of them could have an important impact on human health, such as chlamydophilosis (36), campylobacteriosis (37), and salmonellosis (38). Parrot fever (chlamydophilosis), which is caused by intracellular bacteria, *Chlamydia psittaci*, lives within the respiratory system of birds. Inhalation of dust, dander, and nasal secretions of infected birds is the main way of transmission to humans (39, 40). The mild to severe flu-like illnesses may develop and infected people might be misdiagnosed as influenza.

There is unfortunately a lack of quantitative research into the antimicrobial susceptibility of bacterial zoonotic organisms isolated from bite/scratch wounds or companion animal associated gastroenteritis. Zambori et al. (5) revealed an increased prevalence of drug resistance in animal bite isolates from people. Furthermore, methicillin-resistant *Staphylococcus aureus* (MRSA) or extended-spectrum beta-lactamases (ESBL) producing Enterobacteriaceae, which are known as nosocomial infections have been frequently isolated in companion animals (41), including horses (42). It might be one of the main reasons for the rising prevalence of these potential zoonotic pathogens in human clinical samples.

## Farm Animal-Borne Bacterial Zoonoses

Food producing animals in stock has reached a total of more than 200 million (cattle, pigs, sheep, goats, and chicken) living on farms in Europe (see text footnote 1). It has been demonstrated that farm animals are reservoirs of many zoonotic pathogens to humans (34, 43). However, annually, a large amount of drugs are being used worldwide to sufficient quantities of food to feed a rapidly growing world human population (44–47). The farm animals consume worldwide approximately eight million kilograms of antibiotics annually (70% of which is used for non-therapeutic purposes such as growth promotion; forbidden in the EU from January 2006, and disease prevention) compared with only approximately one million kilogram per year used in human medicine (7). Antibiotics are routinely fed to livestock as growth promoters to increase profits and to ward off potential bacterial infections in the stressed and crowded livestock factory environment (48–52).

Despite large differences in methodology, most results demonstrate that not so long after the introduction of an antibiotic in veterinary practice, resistance in pathogenic zoonotic bacteria and/or the fecal flora increases. In particular, the wide-spread use of antibiotics in animals has resulted in an increased emergence of bacterial resistance to antibiotics, in zoonotic organisms such as *Salmonella, Campylobacter, Shigella, Yersinia, Listeria*, and *Enterococcus* genera, as well as the *E. coli* species. These zoonotic bacterial organisms are propagated primarily among animals and subsequently infect people (53–56). Humans can be infected by contact with animals and their dung or droppings or consumption of infected food. Severe diarrhea may develop, sometimes with blood in the feces. At all ages, but especially in children under 5 years and adults over 65 years, very serious illnesses often occur. These complications can result in loss of life or permanent kidney damage. According to the latest epidemiological trends, *Salmonellosis* and *Campylobacteriosis* are indicated as the most frequent food-borne bacterial zoonoses in Europe. The main food sources were eggs and mixed foods (57).

Furthermore, the recent emergence of ESBL-producing and carbapenemase positive Enterobacteriaceae bacteria in animal production (58), the emergence of farm associated MRSA ST398 (the main pig associated clone) (59–61), and of plasmid-mediated quinolone resistance in animal isolates and food products (62, 63) are great threat for public health. Unfortunately, these antimicrobial resistant “superbugs” are not only confined to hospital environments where antimicrobial use was high and many pathogens were prevalent. They are already widespread in the European community and animal populations that have a great hazard on public health (64, 65).

The causative agent of bovine tuberculosis, *Mycobacterium bovis* (*M. bovis*) has been identified worldwide. Thanks to decades of disease control measures that the occurrence of the infection has been greatly reduced. But there are still hundreds of new cases of human tuberculosis reported in the USA (66). Experience in Europe and the USA, has shown that *M. bovis* can be controlled when it is restricted in livestock; however, eradication is almost impossible once it has spread into wildlife as follows; the European badger in the United Kingdom (67), elk in Canada (68) and white tailed deer in the USA (69).

In the last decade, Q fever caused by *Coxiella burnetii* was one of the most devastating farm animal originated bacterial zoonotic bacteria in Europe. The Netherlands, in particular, has experienced several outbreaks from 2007 to 2010 following identification of a Q fever outbreak on various dairy farms in 2007. Infected humans were mainly located within the intensive dairy goat farms (<5 km) (70). The infection is spread by ticks, inhalation of the organism from the placental fluids, urine, and consumption of unpasteurized milk – meat products of sheep, goats, and cattle. The clinical signs in humans might be severe flu-like syndrome that may last for months (71).

## Vector-Borne Bacterial Zoonoses

In the EU, many vector-borne zoonotic diseases are considered as emerging infectious diseases, which either appear in a population for the first time or may have existed previously but spreading rapidly. The ecology of vector-borne zoonotic bacterial diseases is complex where climate and weather may influence the transmission cycles. Milder winters, earlier start of spring or long intervals between winters cause extended seasonal tick activity and hence pathogen transmission between hosts in new regions of the world (72, 73). Many vector-borne infections occurred in new regions in the past two decades, while many endemic diseases have increased in incidence (74).

The following bacterial pathogens were most frequently identified as the causes of emerging vector-borne infections in the last decades in the EU: *Rickettsiae* spp., *Anaplasma phagocytophilum, Borrelia burgdorferi, Bartonella* spp., and *Francisella tularensis* (75, 76).

*Rickettsia rickettsii* causes Rocky Mountain spotted fever and spreads to humans by ticks. The signs of this disease are fever, headache, muscle pain, and spots with very dark rash. Hiking in an area with many infested ticks is a great risk factor. A tick bite of <20 h is usually not enough to transfer these bacteria to a person (77).

Ehrlichiosis *(Anaplasma phagocytophilum)* and Lyme disease (*Borrelia burgdorferi*) have emerged as an important vector-borne zoonotic disease since 1980s (78, 79). Hard ticks are principal vectors, whereas small rodents are known as their natural vertebrate reservoir. A wide variety of signs including rash, joint pains, fever, enlarged lymph nodes, and some neurological signs may develop. The trend of house buildings in woodlots where humans share the same ecology with reservoirs and vectors was found to be correlated with the increased frequency of such diseases in humans (79).

*Bartonella* spp. is transferred to humans via fleas, lice, and sand flies (80). However, recent studies have shown the importance of tick exposure in human bartonellosis (81). As previously mentioned elsewhere in this article, bartonellosis are usually associated with cat-scratch diseases. Lately, researchers have revealed that *Bartonella* spp. can be transmitted via cat fleas without any scratches to humans (82). Symptoms include fever, enlarged lymph nodes (after 1–3 weeks), and a papule at the inoculation site.

Etiological agent of tularemia, *F. tularensis*, is a rare disease in Europe (83). Bacteria are usually transferred by slaughtering (hunters are at a higher risk), eating of infected hares, respiration of dust, or drinking of contaminated water (84). The prevalence of *F. tularensis* was found to be 1–5% from dog ticks in North America (85). Clinical symptoms depend on how the organism is acquired: erythematous papule at inoculation side within 48 h, pneumonia (the most serious form), endotoxemia, which gives continuous fever, acute pharyngotonsillitis, mucopurulent conjunctivitis (rarest form) (86).

Among many others, brucellosis, which is not an emerging disease, has a significant impact on the endemic Southern European countries with sporadic outbreaks. Fortunately, the impact on humans has not increased since 2000 (87). However, the cross border tracing of some *Brucella* strains isolated in Germany revealed concordance with sheep isolates originating from Eastern Anatolian, Turkey. It is a characteristic example for the global spread of such diseases, in that case most probably by Turkish immigrants living in Germany (88).

Plague, caused by *Yersinia pestis*, is the most important re-emergent bacterial wild rodent borne disease. The current case reports of plague are primarily limited to Africa. However, it is a great potential public health hazard for Europe due to increased traveler mobility or a potential bioterrorist attack (89).

## Discussion and Conclusion

Bacterial zoonoses have a major impact on global public health. Both emerging and re-emerging bacterial zoonoses have gained increasing national and international attention in recent years. The closer contact with companion animals and rapid socioeconomic changes in food production system has increased the number of animal-borne bacterial zoonoses.

Animal bite injuries in daily human-animal contact are not surprising, especially for the school-aged children. Most of these wounds are medicated by patients as first aid and not registered in health systems. In more developed countries, most of the victims with moderate to severe bite injuries will seek professional medical treatment. Regardless, all bites should be treated as serious, especially if the skin is broken. Prompt diagnostic and treatment can prevent wound complications. The possibility to form biofilms by previously mentioned wound microorganisms is quite high, may cause severe tissue damage and protect the bacteria from innate-immune response and antimicrobials. The most of the commercial topical agents and wound dressings are ineffective against the biofilm matrix. Surgical repair (for example, CO_2_ surgical laser techniques, Leon Cantas, personal research notes 2014), which is usually used to obtain a better cosmetic result might be needed to remove biofilm formed bite infections. This mechanical debridement is essential in the eradication of a wound biofilm. Antimicrobials may be more effective in the treatment of the wound after debridement in the prevention of biofilm reformation. Despite the use of currently optimal culturing methods, approximately 7% of infected wounds yield no bacterial growth. In such cases, some other fastidious pathogens, i.e., *Chlamydia* spp., *Mycoplasma* spp., and even viruses should be investigated. New advanced molecular diagnostic techniques are needed. Prevention strategies for animal bites include close supervision of child–animal interactions, stronger animal control laws, better reporting of animal bites, and public education for better ownership of pets. Regular nail trimming, routine oral examinations under annual health checks and comprehensive dental treatments of the companion animals (i.e., routine removal of the teeth tartar and plaques) by veterinarians will reduce the bacterial mass exposure to humans in case of direct contacts or animal bites.

It is important to realize that enteropathogenic zoonoses may be contracted from both clinically sick and apparently healthy companion animals. Feeding of pets with raw food diets is a potential source of *Salmonella, Campylobacter*, and other important bacterial zoonoses; however, some recalls of commercial pet food diets have also occurred as a result of contamination with those microorganisms. Pig ear dog treats, in particular, have been implicated as an important source of *Salmonella* infection for dogs, which can also serve as a source of infection to humans.

Nevertheless, it can be said that easy-to-use personal hygiene rules should be applied by companion animal owners. Thorough hand washing with soap after handling of a companion animal and before eating or drinking, avoiding mouth-to-mouth contact, avoiding aerosolization of dusty fecal matter will help to prevent transmission of the zoonotic disease to humans. The animals with diarrhea should be isolated immediately and veterinary advice should be sought. The household should be cleaned with agents and kept as clean as possible.

On the other hand, the prevalence of antimicrobial resistance in small animal pathogens is increasing globally due to overuse of broad spectrum antibiotics by veterinarians. There is an immediate need for worldwide smarter use of antimicrobials that have some positive effect on the recovery of animals from life threatening diseases. National veterinary antimicrobial treatment guidelines should be established by the local authorities according to the updated routine surveillance results.

Chronic diarrhea, dermatitis, ear and eye infections of pets caused by microbes demand longer durations of antimicrobial remedies at home. More frequent use of advanced laboratory tests, such as; feed/insect/mould allergy tests and differential diagnosis of the other relevant auto-immune disorders may help to investigate the main underlying cause of the such reactions which can be managed in various alternative treatment methods (i.e., hypoallergenic diets) rather than antibiotics solely. Herein, pet specific auto-immune vaccines against allergens and auto-Lactobacillales (Auto-Lac, Leon Cantas, personal research notes, 2011–2014) as dietary supplements can also be more frequently administered within the preventative veterinary practice measures. Owners should be encouraged to insure their family animals to afford such costly veterinary services contradictory to the cheaper and sometimes life-long medical (i.e., antibiotic) treatment demanding options. Veterinarians should also spear more time to educate the pet owners under consultations to handle infected-antimicrobial treated animals with precaution due to irreversible consequences of the antimicrobial resistance development and its spread in households. Proper hand washing and use of gloves are strictly recommended while handling antimicrobial in veterinary clinics. Veterinarians should prescribe broad spectrum and synthetic antimicrobials preferably after culturing with extreme precautions (i.e., dosage, dosing intervals and length of the treatment). Reduced antibiotic use will hinder the development of antibiotic resistance in animal microbiota which might cause zoonotic infections in humans (50, 52).

Food-borne zoonoses are an important public health concern worldwide and every year a large number of people affected by diseases due to contaminated animal originated food consumption. Food hygiene education of the consumers is an important competent of food-borne diseases prevention. However, main prevention of food-borne zoonoses must begin at the farm level with in the concept of “*One Health*.” Herein, control of the production stress especially in intensive livestock industry, with the development of better animal health management routines (i.e., routine vaccinations, immune stimulants: pre-, probiotic feed additives) and the increased animal welfare programs, will contribute eventually to an optimal production of animal health. Increased antimicrobial resistance among emerging and re-emerging farm-borne bacterial pathogens in crowded settings (i.e., poultry, pig farms) is a growing problem. Restrictive antimicrobial choice with better animal welfare managements are needed to control the spread of antibiotic resistance elements.

In the EU, the use of avoparcin was banned in 1997 and the use of spiramycin, tylosin, and virginiamycin for growth promotion were banned in 1998. All other growth promoters used in feeding of food producing animals were banned from January 1, 2006 after a few national bans the years ahead^3^. In the U.S., politicians are still discussing to introduce a similar ban (S-742, 109th U.S. Congress (Preservation of Antibiotics for Medical Treatment Act). Despite the ban on the use of all antibiotics as growth promoters in the EU and a ban on the use of quinolones as growth promoters in the poultry feed in the US medical, important antibiotics are still routinely fed to livestock prophylactically to increase profits and to ward-off potential bacterial infections in the stressed and crowded livestock and aquaculture environments in some parts of the world (50, 90, 91). Because stress lowers the immune system function in animals, antibiotics are seen as especially useful in intensive animal confinements (92). The non-therapeutic use of antibiotics involves low-level exposure in feed over long periods – an ideal way to enrich resistant bacterial population (93, 94). Moreover, antibiotic resistance has been detected in different aquatic environments (95). Fish pathogenic bacteria often produce devastating infections in fish farms where dense populations of fish are intensively reared. Bacterial infections in fish are regularly treated with antibiotics in medicated feed. So far, most of the fish pathogenic bacteria with a history in diseased fish farms have developed drug resistance (96). Modern fish farming relies increasingly on vaccination procedures and improved management to avoid infections (97). For example, the Norwegian aquaculture industry has produced over one million tons farmed fish^4^ by using improved vaccines, management techniques, and only 649 kg of antimicrobials in 2011 (98).

Vector-borne and zoonotic bacterial pathogens are a major source of emerging diseases, and since the time of Hippocrates, weather and climate are linked to the incidence of such infectious diseases. Complexity of epidemiology and adoptive capacity of microorganisms and the arthropods make the vector-borne disease almost impossible to eradicate. Insect repellants, routine tick checks after outdoor activity in risk regions, prompt-proper tick removal, use of long sleeves and trousers (light-colored), and routine insecticide treatment of pets are recommended as general preventative measures (99). Herein, Lyme disease, tick-borne illness, is vastly underestimated over past decades and clearly the urgent prevention is needed. Besides individual awareness of such vector-borne diseases, better national surveillance and reporting programs will contribute to improved the disease control strategies. Clinicians have an important role in the effective management of vector-borne zoonotic diseases, with enhanced differential diagnostic skills based on clinical symptoms and rapid molecular identification techniques (100–103). Most of the time, the clinicians are on the first line of detection of these epidemics due to large group of patients with novel sets of similar symptoms. Increased medical networking via online databases offer a broad overview to followers with regard to changes in temporal patterns of illness in real time, which helps faster detection of new epidemics (104).

Identification and control of emergent zoonotic bacterial diseases require a “*One Health*” approach, which demands combined efforts of physicians, veterinarians, epidemiologists, public health workers, and urban planners. Collaborative international routine surveillance strategies, prompt – reliable agent identification techniques, and optimization of the treatment regiments will ensure the prevention and management of such infections.

## Author Contributions

Leon Cantas defined the review theme, manuscript design, established the coordination and the collaborations, designed the manuscript, contributed to the data collection, data analysis, and drafting, writing and editing of the manuscript. Kaya Suer contributed to drafting and editing of the manuscript. All authors have seen, and approved the manuscript.

## Conflict of Interest Statement

The authors declare that the research was conducted in the absence of any commercial or financial relationships that could be construed as a potential conflict of interest.

## Footnotes

^1^http://epp.eurostat.ec.europa.eu/

^2^http://www.fediaf.org/facts-figures/

^3^http://europa.eu

^4^http://www.ssb.no/fiskeoppdrett_en/
